# Probiotic Lactic Acid Bacteria-Fermented Beverages from Bambara Groundnut and Cowpea Sprouts Modulate Gut Microbiota and Short-Chain Fatty Acids

**DOI:** 10.3390/foods15071141

**Published:** 2026-03-26

**Authors:** Nobahle Pretty Cele, Yusuf Olamide Kewuyemi, Oladipupo Adiamo, Eshetu Mulisa Bobasa, Jiale Zhang, Maral Seididamyeh, Yasmina F. Sultanbawa, Dharini Sivakumar

**Affiliations:** 1 Phytochemical Food Network, Department of Crop Sciences, Tshwane University of Technology, Pretoria 0001, South Africa; zamandoccy24@gmail.com (N.P.C.);; 2 Centre for Nutrition & Food Sciences, Queensland Alliance for Agriculture and Food Innovation, The University of Queensland, Brisbane, QLD 4108, Australia; o.adiamo@uq.edu.au (O.A.); e.bobasa@uq.edu.au (E.M.B.); jiale.zhang1@student.uq.edu.au (J.Z.); s.maral@uq.edu.au (M.S.); y.sultanbawa@uq.edu.au (Y.F.S.)

**Keywords:** synbiotic foods, legume-based beverages, in vitro gut model, microbial ecology, *Bifidobacteriaceae*, *Lactobacillaceae*

## Abstract

Underutilised, nutrient-dense legumes in their sprouted form provide promising substrates for developing functional fermented foods capable of influencing gut microbial activity and metabolite production. This study evaluated the effects of probiotic lactic acid bacteria-fermented beverages derived from sprouted Bambara groundnut (*Vigna subterranea*) and cowpea (*Vigna unguiculata*) on gut microbiota composition and short-chain fatty acid (SCFA) production using an in vitro colonic fermentation model. The beverages were fermented with either *Bifidobacterium animalis* BB-12 (BCBF24) or *Lactiplantibacillus plantarum* 75 (BCL7524). During colonic fermentation, at 0, 12, 24, and 38 h, faecal slurries were collected for SCFA analysis using gas chromatography–mass spectrometry (GC-MS) and deoxyribonucleic acid (DNA) sequencing (Oxford Nanopore Technologies). Microbial diversity decreased, indicating selective enrichment of taxa. BCL7524 induced a major shift, significantly (*p* < 0.05) enriching *Bacillota* and driving *Megasphaera* to ~42% dominance within 24 h. This reflected cross-feeding from *L. plantarum* to lactate-utilising *Megasphaera* spp. Spearman correlation linked *Megasphaera* to a broad SCFA profile, including isobutyric, isovaleric, valeric, and hexanoic acids, with a significant (*p* < 0.05) positive correlation observed for hexanoic acid. Kyoto Encyclopedia of Genes and Genomes (KEGG) analysis indicated proteolysis and mapped hexanoic acid to fatty acid biosynthesis pathways, suggesting chain-elongation activity contributing to hexanoate formation. In line with this, BCL7524 produced significantly (*p* < 0.05) higher levels of hexanoate (3–14 mM) and valerate (10–15 mM), supporting chain-elongation activity within the community. In contrast, BCBF24 enriched *Actinomycetota* and *Bifidobacterium*, correlating with acetate production (18–23 mM). This study demonstrates that specific synbiotic beverages can modulate gut microbial ecology and metabolic output under in vitro conditions.

## 1. Introduction

Bambara groundnuts (*Vigna subterranea* (L.) Verdc.) and cowpeas (*Vigna unguiculata* (L.) Walp.) are ideal and affordable substrates for non-dairy beverages. These legumes are rich sources of proteins, fibre, vitamins (niacin, thiamine, tocopherol, folate), minerals (calcium, iron, magnesium, and zinc), and bioactive compounds [1] and contribute to various health benefits, including antioxidant, anti-inflammatory, and anti-diabetic effects, which support their role in disease prevention and nutritional well-being [1,2]. As climate-resilient crops, they play a vital role in a resilient food security diet [3]. Bambara groundnut and cowpea remain underutilised industrially, due to antinutritional factors and storage-related defects such as hard-to-cook phenomena, which hinder processing and reduce nutrient bioavailability [3,4,5]. Despite bioprocessing techniques reducing these issues, few studies have explored the transformation of underutilised legumes into consumer-acceptable functional products.

A variety of processing strategies, such as germination (sprouting) [6] and lactic acid bacteria (LAB) fermentation, enhance legume functionality through essential metabolic processes [7]. Probiotics delivered through plant-based non-dairy beverages can meet the growing demand for lactose-free and vegan functional foods [8]. Germination reduces antinutritional factors and increases proteins, dietary fibre, and bioactive compounds in Bambara groundnut and cowpea [6,9]. LAB fermentation enhances antioxidant activity of sprouted legume-based beverages by adding vitamins, organic acids, and phenolic compounds [4]. Further, these bioprocessing techniques transform legumes into synbiotic systems capable of delivering both prebiotics and probiotics to modulate gut microbiota [10,11]. In order for legume-based synbiotic beverages to confer health benefits, their bioactive components must survive digestion, be metabolised, and interact with gut microbiota [12]. Unabsorbed food components, particularly dietary fibres, are fermented by the gut microbiota to produce short-chain fatty acids (SCFAs), which play a critical role in metabolism and immunity [13]. The consumption of synbiotic beverages can improve the metabolic function of the gut microbiome [10].

In vitro digestion and colonic fermentation models provide controlled platforms for assessing bioaccessibility, microbial community shifts, and fermentation metabolites [12,14]. However, the impact of in vitro colonic fermentation of synbiotic beverages derived from traditional legumes, sprouted Bambara groundnut and cowpea remains largely unexplored.

The nutritional profile of indigenous legumes such as Bambara groundnuts and cowpeas can be significantly enhanced by optimising sprouting durations while also reducing antinutritional compounds [6]. Given these findings, this study aimed to investigate the in vitro colonic fermentation of a novel LAB-fermented synbiotic beverage produced from sprouted Bambara groundnut and cowpea to understand their capacity to modulate gut microbiota composition and influence SCFA production. This knowledge is crucial for developing effective plant-based synbiotic beverages that meet consumer demand for convenient, health-promoting functional foods.

## 2. Materials and Methods

### 2.1. Materials

Bambara groundnut seeds with brown, cream, and red seed coats, as well as cowpea seeds with brown and cream seed coats, were procured from NTK Pietersburg, Limpopo, South Africa. Commercial soymilk (Loftus Park, Pretoria, South Africa) served as the reference sample. Analytical-grade reagents and chemicals were used in this study unless otherwise indicated.

### 2.2. Sample Preparation and Formulation

Bambara groundnut and cowpea sprouts were prepared according to Cele et al. [6]. Based on their findings that sprouting times of 48 h for Bambara groundnut and 24 h for cowpea, along with specific genotypes (red, brown, and cream), yielded higher protein content, these durations and genotypes were adopted for the present experiment. After sprouting, Bambara groundnut genotypes in a ratio of 40% red: 40% brown: 20% cream, and cowpea genotypes in an equal 50% brown: 50% cream ratio were individually combined. Each composite sprouted mix was separately blended with 10% (*w*/*v*) water to extract the pulse-based milks, followed by mixing. The extracted Bambara groundnut and cowpea milks at a 1:1 ratio were pasteurised (WBE28A12E, PolyScience, Niles, IL, USA) at 60 ± 2 °C for 30 min.

### 2.3. Production of LAB-Fermented Synbiotic Beverages

*Lactiplantibacillus plantarum* 75 and *Bifidobacterium animalis* subsp. *lactis* BB-12 were reactivated and incubated in de Man, Rogosa and Sharpe (MRS) broth at 30 °C for 48 h under anaerobic conditions [15]. The harvested cells were washed twice with sterile distilled water via centrifugation (MSE, Harrier 15/80, Sydenham, London, UK) (1000× *g*, 10 min) and resuspended to an initial optical density (OD_720_) of 0.005, corresponding to 1–5 × 10^8^ CFU/mL per McFarland standards. This inoculum was stored at −4 °C overnight before use. The pasteurised pulse-based milk blend was inoculated with 1% (*v*/*v*) of each LAB starter and fermented in opaque bottles at 37 °C for 24 h. The resulting fermented beverages, BCL7524 (with *L. plantarum* 75) and BCBF24 (with *B. animalis* subsp. *lactis* BB-12), and soymilk powder served as the reference sample for all subsequent analyses.

### 2.4. Chemical and Microbiological Analyses

Total dietary fibre (TDF) content was determined using the TDF assay kit from Megazyme (Bray Business Park, Bray, Co., Ltd., Wicklow, Ireland), according to the AOAC 991.43 method [16].

The total counts of viable LAB were determined according to the method described by Cele et al. [17], with minor modifications. Exactly 1 mL of the sample was homogenised with 9 mL of 0.85% (*w*/*v*) sterile saline solution. This initial mixture was serially diluted to obtain a suitable bacterial population. Aliquots (100 µL) of the diluted samples were spread onto MRS agar. The inoculated plates were then incubated aerobically at 37 °C for 48 h. After the incubation period, the resultant colonies were counted to monitor LAB survival and viability was expressed as Log colony-forming units per millilitre (Log CFU/mL) of milk (Table S1).

The total phenolic content (TPC) and ferric reducing antioxidant power (FRAP) of the samples were determined using the procedure by Adiamo et al. [14]. The TPC was expressed as milligrams of gallic acid equivalent per gram (mg GAE/g) and the FRAP recorded as micromoles of ferrous equivalent per gram (µmol Fe^2+^Eq/g).

### 2.5. Gut Dynamic Model System Analysis

#### 2.5.1. Faecal Slurry Preparation and Stabilisation

Approval for this study was obtained from the University of Queensland’s Human Research Ethics Committee (2021/HE001428). Fresh faecal samples were collected from three healthy volunteers, all of whom consented to participate in the experiment. Eligible donors (average age = 32) had not consumed probiotics, antibiotics, or phenolic supplements in the last six months. The experiments were conducted in full compliance with the university’s ethical guidelines. Faecal samples were pooled to generate a representative inoculum, and the resulting slurry was prepared and stabilised according to the protocol reported by Molly et al. [18], as revised by Gaisawat et al. [19].

#### 2.5.2. Upper Gastrointestinal (GI) Digestion and Colonic Fermentation

On the experimental day, 90 mL of saline buffer (0.9% NaCl, pH = 7.4) was added to 250 mL reaction bottles containing 10 g of samples, i.e., four treatment groups: BCBF24, BCL7524, soymilk and a control group (saline buffer only). The mixture was stirred using a magnetic stirrer (Thermo Fisher Scientific, Waltham, MA, USA) at low speed throughout the experiment. Oral (pH 7.0 for a period of 15 min), stomach (pH 2.0 for 1.5 h), and small intestine (SI) (pH 8.0 for 2 h) digestion in each bottle were stimulated by adding 2 mL α-amylase (3000 U/mL) (A3176, Merck Life Science Pty Ltd., Bayswater, VIC, Australia), 2 mL pepsin (1000 U/mL of pepsin) (P7125, Merck Life Science Pty Ltd., Bayswater, VIC, Australia) and 10 mL pancreatic juice (12 g/L NaHCO_3_, 6 g/L bile extract, and 0.9 g/L pancreatin), respectively. Finally, 50 mL of the overnight stabilised faecal slurry was added to each bottle to mimic the colon fermentation. The fermentation was conducted under anaerobic conditions at pH 6.2 for a 38 h period. Approximately 20–30 mL of solution was collected at 0, 12, 24, and 38 h of fermentation, followed by centrifuging for 10 min at 2000× *g* using Thermo Scientific Multifuge (Thermo Fisher Scientific, Waltham, MA, USA). The supernatant was passed through sterile 0.2 μm syringe filter membranes, whereas the pellet was also retained for gut microbiota composition analysis. Both the filtered samples and pellets were stored in −80 °C freezer until analysis. The experiment (Figure S1) was conducted in three biological replicates.

### 2.6. Deoxyribonucleic Acid (DNA) Extraction and Sequencing

DNA extraction was referred to as the method of QIAamp^®^ PowerFecal^®^ Pro DNA Kit (QIAGEN, Venlo, The Netherlands). The DNA quality and concentrations were measured using NanoDrop Spectrophotometer (Thermo Fisher Scientific, Waltham, MA, USA) and Qubit fluorometer (Thermo Fisher Scientific, Waltham, MA, USA), respectively. Extracted DNA was used for library preparation and sequencing using the Ligation Sequencing gDNA—Native Barcoding Kit 24 V14 (Oxford Nanopore Technologies, Oxford, UK). Further, sequencing was performed on an Oxford Nanopore MinION sequencer using a MinION R10.4.1 flow cell (FLO-MIN114, Oxford Nanopore Technologies, Oxford, UK) for 72 h. Oxford Nanopore Technologies EPI2ME software was used for post-data analysis. Standard-8 database (the default for the Kraken2 workflow), which contains whole genome sequences, was used for taxonomical classification. In the Results and Discussion Section, updated phylum-level nomenclature (e.g., *Bacillota*, *Bacteroidota*, *Actinomycetota*) is used in accordance with recent taxonomic reclassification. Where earlier studies refer to legacy phyla such as ‘*Bacteroidetes*’ and ‘*Actinobacteria*,’ these terms correspond to the updated phyla *Bacteroidota* and *Actinomycetota*, respectively.

### 2.7. Identification and Quantification of Short-Chain Fatty Acids (SCFA) Using Gas Chromatography–Mass Spectrometry (GC-MS)

The digesta samples (200 µL) and a 200 μL aliquot of ice-cold internal standard of 2-methyl hexanoic acid (0.1 mg/mL prepared in 0.2 M NaOH) were mixed in 15 mL Falcon tubes. Then, 400 μL of a mixture of benzyl alcohol and pyridine (BnOH–Py) at a ratio of 3:2 (*v*/*v*) was added to the mixture, followed by adding 200 μL of DMSO, and vortexed for 5 s until a clear solution was obtained. The mixture was derivatised by adding 200 μL benzyl chloroformate with gentle vortexing for 3 min at room temperature. The derivatised SCFAs were extracted by adding 800 μL cyclohexane, vortexed at low speed, and centrifuged (Thermo Fisher Scientific, Waltham, MA, USA) (4000 rpm, 4 °C, 5 min). Thereafter, the 200 μL cyclohexane layer was transferred into a new vial for analysis using a Thermo Scientific™ ISQ 7000 GC-MS system (Thermo Scientific, Waltham, MA, USA). Short-chain fatty acid benzyl ester derivatives were separated using an Agilent HP-INNOWax capillary column (30 m × 0.25 mm id, 0.25 μm; Agilent Technologies, Mulgrave, VIC, Australia). The injection volume was 1 µL, a split mode ratio of 20:1 was employed, and helium served as the carrier gas with a flow rate of 1.2 mL/min. The temperatures were configured as follows: 250 °C for the front inlet, 250 °C for the transfer line, and 230 °C for the ion source. The temperature gradient programme for the column started at 70 °C and was maintained for 3 min and then increased at a rate of 10 °C/min to 200 °C, followed by a rise to 240 °C at 35 °C/min, where it remained steady for 7 min. Chromeleon ver. 7.2 and Trace Finder ver. 5.1 software were employed for data acquisition and data processing, respectively. The SCFA mix standard solution (46975-U, Merck Life Science Pty Ltd., Bayswater, VIC, Australia) was derivatised as well and used to plot the standard curve and their mass list is presented in Table S2. The SCFA results were reported as individual in each digesta. The analysis was conducted in duplicate for each fermentation replication.

### 2.8. Statistical Analysis

The statistical analysis was conducted using R 4.5.1. The beta diversity of samples was calculated using Bray–Curtis dissimilarities and visualised using principal coordinate analysis (PCoA). Tukey’s honest significant difference (HSD) post hoc test was applied following a one-way ANOVA to identify significant differences among the means. For taxonomical analysis, phylum-level read counts were converted to relative abundance (%) per sample. The three most abundant phyla were identified based on overall mean abundance and analysed using one-way ANOVA with Tukey’s HSD for (i) substrate comparisons within each time point and (ii) time comparisons within each substrate. Significant pairwise differences were visualised using bracket annotations with adjusted *p*-value significant levels. SCFAs and gut microbiota were correlated using Spearman correlation. KO identifiers were mapped using the KEGG REST API, implemented with the KEGGREST package [20] to identify pathways.

## 3. Results and Discussion

### 3.1. Microbial Community Diversity

**Figure 1:**
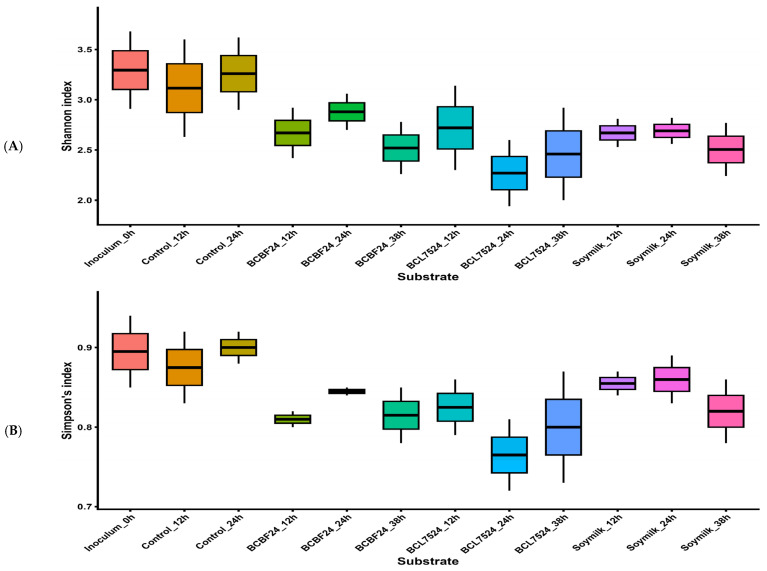

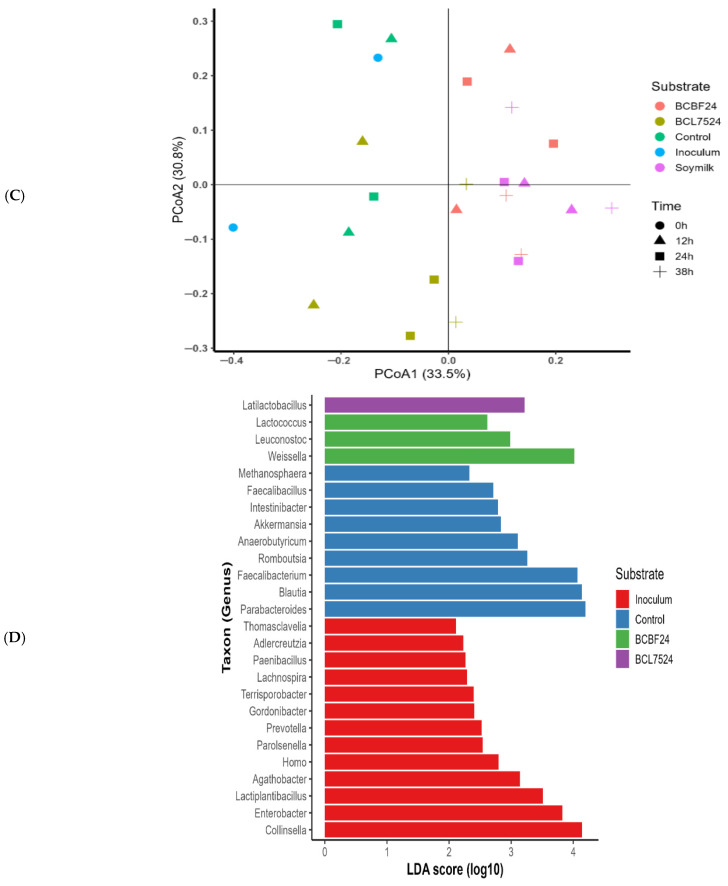
Microbial community diversity following in vitro colonic fermentation of the different substrates. (**A**) Shannon index; (**B**) Simpson index; (**C**) Principal coordinate analysis (PCoA) of beta diversity based on Bray–Curtis dissimilarity; and (**D**) linear discriminant analysis effect size (LEfSe; LDA > 2). No significant differences were observed among substrate types for alpha diversity of the microbial community structure. Treatments: control (saline buffer only), BCBF24 (sprouted Bambara groundnut-cowpea milk fermented with *Bifidobacterium animalis* subsp. *lactis* BB-12), BCL7524 (sprouted Bambara groundnut-cowpea milk fermented with *Lactiplantibacillus plantarum* 75), inoculum, and soymilk.

The Shannon index, which measures community evenness, and Simpson’s index, which measures dominance, were used to evaluate alpha diversity [21]. In vitro colonic fermentation over 38 h results in changes in alpha diversity across substrates (Figure 1A,B). The control group maintained a relatively high and stable diversity (Shannon: 3.1–3.3, Simpson: 0.87–0.9) compared with the beverage powder treatments, indicating that microbial diversity was maintained in the saline buffer control. Both the synbiotic milk and soymilk treatments decreased microbial diversity following inoculation. The BCL7524 treatment induced the most pronounced decline. After 24 h, Shannon and Simpson’s indices reached their lowest median values (approximately 2.3 and 0.76, respectively), suggesting a substantial decrease in diversity (Figure 1A,B). This sharp decline indicates selective enrichment, likely due to *L. plantarum*’s competitive growth advantage in producing lactic acid, which lowered pH and probably released phenolic aglycones [22] (Figure S2). BCL7524 may have stimulated a limited range of bacterial species, resulting in LAB dominance (7.33 Log CFU/mL) (Table S1). Such a marked drop in diversity may reflect a targeted fermentation process where specific beneficial microorganisms are promoted to achieve defined metabolic outcomes [23].

In contrast, BCBF24 also decreased diversity compared with the inoculum and control, but its median in Shannon and Simpson’s indices remained higher than those for BCL7524, particularly at 24 h. This pattern suggests a milder selective pressure, possibly due to lower lactic acid production. The soymilk-maintained diversity levels were similar to, or slightly below, BCBF24, but consistently higher than the lowest point observed for BCL7524. This implies that BCL7524 exerted the strongest influence on microbial composition throughout fermentation.

While alpha diversity measures within-sample variation, beta-diversity analysis reveals microbial compositional similarity among groups. A PCoA based on Bray–Curtis dissimilarity explained 64.3% of the total variance on the first two axes (PCoA1: 33.5%; PCoA2: 30.8%) (Figure 1C). This distinct clustering demonstrates that substrate type altered the microbial ecology.

Samples from BCL7524 exhibited the most distinct shift from the inoculum and control, clustering along the lower negative axis of PCoA1, particularly at 24 h. This separation aligns with the observed reduction in alpha diversity (Figure 1A,B). BCBF24 samples clustered mainly in the upper positive quadrant of PCoA1 and overlapped partially with soymilk samples at later time points. The clear separations highlight that each substrate distinctly reshaped the gut microbial community [23]. These findings suggest that different substrates can shape microbial community structure in vitro, reflecting the potential for modulation under controlled in vivo conditions.

To identify taxa responsible for these shifts, a linear discriminant analysis effect size (LEfSe) analysis was performed (Figure 1D). The initial inoculum displayed a diverse array of differential genera, including *Lactiplantibacillus*, representing the baseline faecal microbiota. High LDA scores for many of these taxa indicate their role as defining features of the starting community [24]. The control group showed significant enrichment of genera, such as *Parabacteroides*, *Blautia*, *Faecalibacterium*, *Romboutsia*, *Anaerobutyricum*, *Akkermansia*, *Intestinibacter*, *Faecalibacillus*, and *Methanosphaera*, supporting earlier observations of its relatively high alpha diversity (Figure 1A,B).

In contrast, BCL7524 was strongly characterised by enrichment of *Latilactobacillus*. This genus, along with *Lactiplantibacillus*, includes species formerly classified under the broad *Lactobacillus* group. The pronounced enrichment of *Latilactobacillus*, with its high LDA score, confirms successful establishment and dominant activity of the inoculated probiotic *Lactiplantibacillus plantarum* 75. This finding agrees with Mastrolonardo et al. [23], who reported that fermented protein improved the relative abundance of some genera like *Lactiplantibacillus*, stimulating SCFA production. Furthermore, *Latilactobacillus* species such as *L. curvatus* and *L. sakei* exhibit antioxidant and anti-inflammatory properties [25]. Thus, the high relative abundance of *Latilactobacillus* in BCL7524 reflects substrate-driven modulation of the microbial community under in vitro conditions, which may indicate the need for further investigation in vivo to determine their physiological relevance.

BCBF24 displayed a different pattern (Figure 1D). Although *Bifidobacterium* was not a top differentiating taxon, this treatment was marked by enrichment of other LAB genera (*Weissella*, *Leuconostoc*, and *Lactococcus*). The high LDA score for *Weissella* highlights that it is the main differential genus in this group. *Weissella* has been linked to the release of bioactive peptides with potential angiotensin-converting enzyme-inhibitory and antioxidant activities [23]. These observations illustrate how different synbiotic formulations can uniquely modulate gut microbiota, generating distinct microbial consortia and metabolic profiles [23,26].

### 3.2. Gut Microbiota Compositional Shifts at the Phylum Level

**Figure 2:**
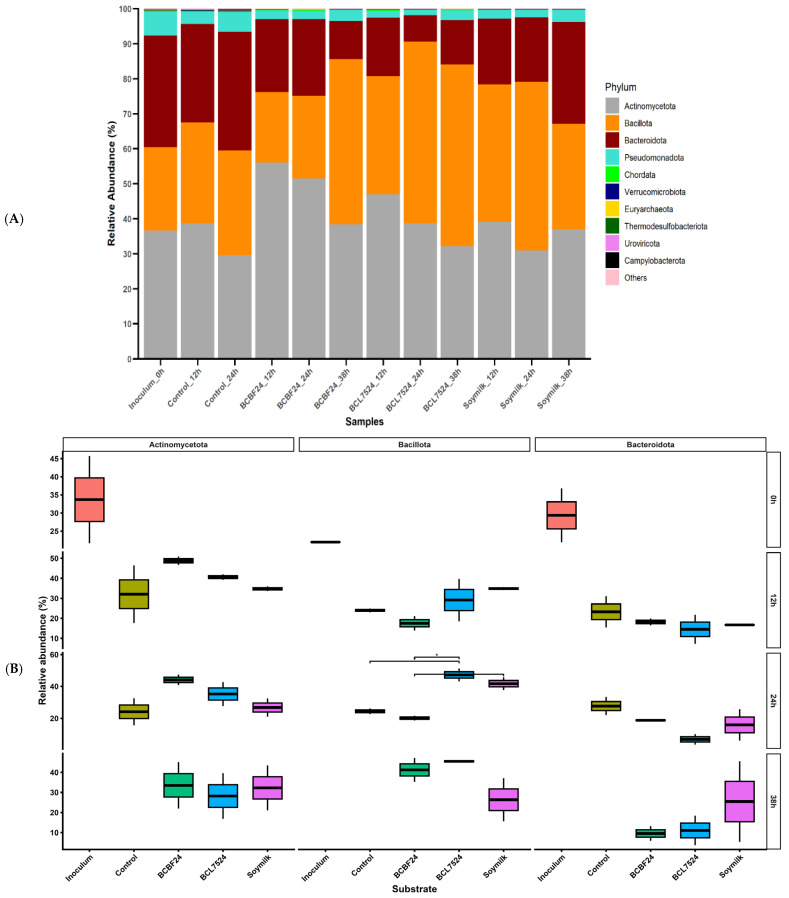

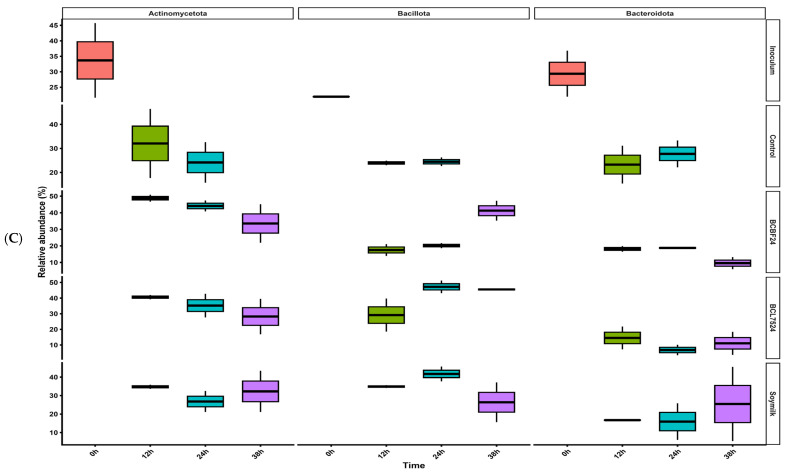
(**A**) Microbial community structure of the top 10 bacterial phyla after 38 h of in vitro fermentation. Treatments: Control (saline buffer only), BCBF24 (sprouted Bambara groundnut–cowpea milk fermented with *Bifidobacterium animalis* subsp. *lactis* BB-12), BCL7524 (sprouted Bambara groundnut–cowpea milk fermented with *Lactiplantibacillus plantarum* 75), inoculum and soymilk. (**B**) Substrate-driven effects on dominant phyla, analysed within each time point (0, 12, 24, 38 h). Significant differences were observed between substrate type for *Bacillota*. * Indicates the observed correlation is significant (*p* < 0.05). Treatments: Control (saline buffer only), BCBF24 (sprouted Bambara groundnut–cowpea milk fermented with *Bifidobacterium animalis* subsp. *lactis* BB-12), BCL7524 (sprouted Bambara groundnut–cowpea milk fermented with *Lactiplantibacillus plantarum* 75), inoculum and soymilk. (**C**) Temporal dynamics of dominant phyla, analysed within each substrate. No significant differences were observed between time points for any individual phylum. Treatments: Control (saline buffer only), BCBF24 (sprouted Bambara groundnut–cowpea milk fermented with *Bifidobacterium animalis* subsp. *lactis* BB-12), BCL7524 (sprouted Bambara groundnut–cowpea milk fermented with *Lactiplantibacillus plantarum* 75), inoculum and soymilk.

The relative abundance of the top 10 bacterial phyla identified in the inoculum, control, synbiotic beverage powders and soymilk substrates during a 38 h period of colonic fermentation is presented in Figure 2A. Across all substrates, *Actinomycetota*, *Bacillota,* and *Bacteroidota* are the predominant gut microbiota composition at the phylum level, which together accounted for over 90% of the total relative abundance.

The synbiotic beverage powders induced a distinctive shift toward *Bacillota* dominance. In the BCBF24 fermentate, *Bacillota* abundance nearly doubled from approximately 24% at 24 h to 48% at 38 h, with corresponding decreases in the relative abundance of *Actinomycetota* and *Bacteroidota*. Similarly, BCL7524 maintained *Bacillota* predominance at approximately 52% relative abundance at both time points. This notable shift suggests that the synbiotic milk provided potent selective enrichment for saccharolytic *Bacillota* native to the gut environment, potentially modulating fermentation pathways toward enhanced SCFA production [27,28]. In contrast, the soymilk exhibited a different successional pattern. *Bacillota* reached approximately 48% at 24 h, and then declined to approximately 30% at 38 h. This was accompanied by increased abundances of *Actinomycetota* and *Bacteroidota*. This observation concurred with the findings of Xiong et al. [29] where the soybean group increased the abundance of *Bacteroidetes* and decreased the abundance of *Actinobacteria*. This divergence indicates that specific components within the synbiotic beverage, rather than general substrate availability, may contribute to being the critical drivers of the sustained *Bacillota* enrichment [23,30].

Analysis within each time point showed that substrate type shaped the relative abundance of the dominant phyla. BCL7524 increased the abundance of *Bacillota* compared with the other substrates with increased fermentation time (Figure 2B). At 24 h, BCL7524 exhibited a significant (*p* < 0.05) increase in *Bacillota* (median: 48%; IQR: 43–52%) compared with the control and BCBF24. Similarly, soymilk showed a significant (*p* < 0.05) increase (median: 42%; IQR: 38–44%) at 24 h compared with BCBF24. The higher abundance observed in BCL7524 may reflect the metabolic activity of *L. plantarum* 75, which lowered the pH and produced lactate, creating a selective environment favouring acid-tolerant and lactate-utilising members of *Bacillota*. Increased *Bacillota* levels corresponded with reduced *Pseudomonadota* abundance (Figure 2A), a phylum associated with characteristic shifts in certain gut microbial imbalances [21].

BCBF24 selectively enriched *Actinomycetota*, reaching a median abundance of approximately 43% (IQR: 40–48%) at 24 h. This was higher than BCL7524 (median: 37%) and soymilk (median: 27%, which resembled the control [median: 25%]) in their *Actinomycetota* profiles (Figure 2B). Although no significant difference (*p* < 0.05) was observed within each time point (0–38 h), this selective enrichment possibly represents a self-reinforcing effect. The inoculated *B. lactis* BB-12, a member of *Actinomycetota*, likely utilised the BCBF24 matrix via the bifidum pathway, producing an acetate-rich environment that supported indigenous *Actinomycetota* without triggering the competitive dominance of lactate-utilising *Bacillota* observed in the BCL7524 fermentate.

Temporal analysis across all substrates revealed consistent successional patterns, with no significant differences (*p* < 0.05) observed between time points for any phylum (Figure 2C). Although these changes were not statistically significant, the overall trends highlight competitive exclusion processes among the dominant phyla. These processes appear to be driven by differences in substrate utilisation and the accumulation of fermentation by-products.

### 3.3. Gut Microbiota Compositional Shifts at the Genus and Species Levels

**Figure 3:**
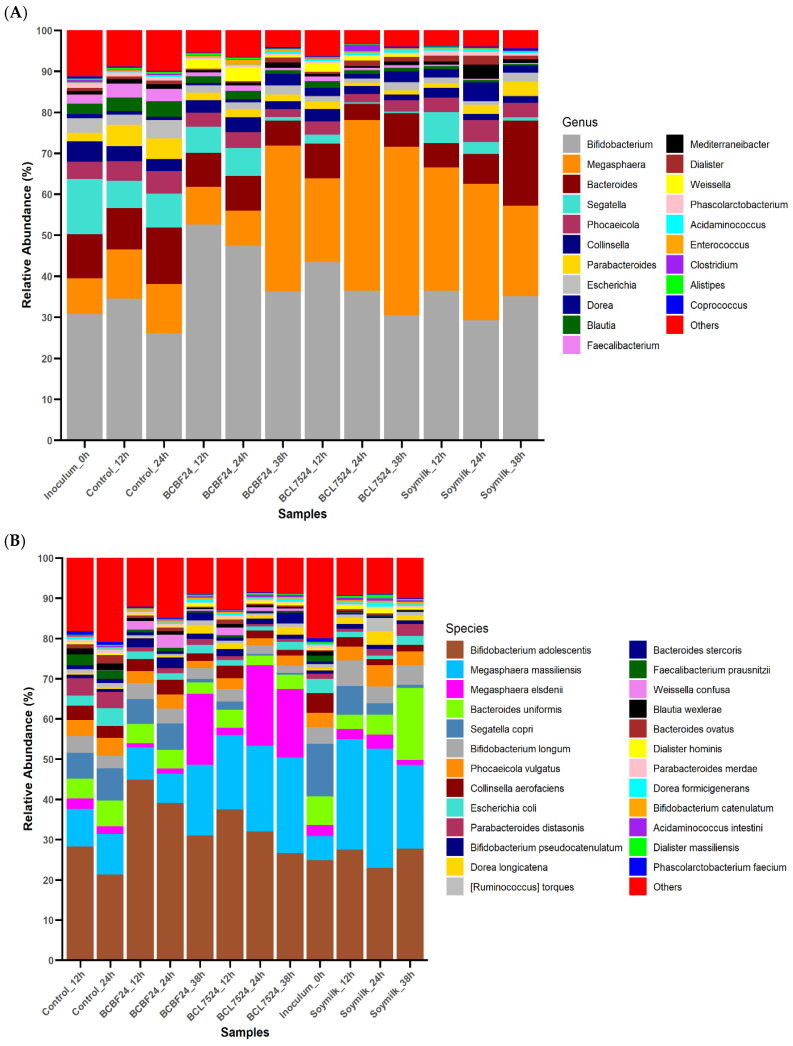
Gut microbiota composition at the genus and species levels after 38 h of fermentation. (**A**) The stacked bar chart shows the relative abundance of the top 20 bacterial genera across different substrates. (**B**) The stacked bar chart shows the relative abundance of the top 25 bacterial species. Treatments: Control (saline buffer only), BCBF24 (sprouted Bambara groundnut–cowpea milk fermented with *Bifidobacterium animalis* subsp. *lactis* BB-12), BCL7524 (sprouted Bambara groundnut–cowpea milk fermented with *Lactiplantibacillus plantarum* 75), inoculum and soymilk.

The gut microbiota composition at genus and species levels during the fermentation of the synbiotic beverage and soymilk, compared to the inoculum and control, is presented in Figure 3. The synbiotic beverage, particularly BCL7524, markedly increased the abundance of the genus *Megasphaera* (Figure 3A). The marked enrichment of *Megasphaera*, a genus known for its ability to utilise lactate [31], supports the premise that the rapid acidification by *L. plantarum* 75 created a niche favourable for acid-tolerant bacteria. It has been proven that *Megasphaera* spp. can synthesise SCFAs to maintain the homeostasis of the intestinal tract [32]. Moreover, the high lactate production by *Lactiplantibacillus* in BCL7524 probably provided a key substrate that further promoted the proliferation of *Megasphaera*. Consequently, a direct ecological succession was observed where *Megasphaera* abundance in BCL7524 increased to approximately 20% within 12 h and became dominant at approximately 42% at 24 h. In BCBF24, this increase was slower, increasing from approximately 8% at 24 h to 35% at 38 h. Species-level analysis revealed that this community shift was primarily driven by *Megasphaera massiliensis* and *M. elsdenii* (Figure 3B). In BCL7524, *M. massiliensis* increased to approximately 24% at 38 h, while *M. elsdenii* increased to approximately 21% at 24 h before a slight decline. The pronounced, more sustained abundance of both *Megasphaera* spp. in BCL7524 indicates that its specific synbiotic matrix was effective at stimulating a cross-feeding relationship. This trend was consistent with a previous study stating that a co-occurrence pattern was found between *Megasphaera* and *Lactobacillus* spp. [33].

However, other lactate utilisers may have been outcompeted and consequently declined in relative abundance. For example, alongside the increase in *Megasphaera*, the synbiotic powders reduced the relative abundance of other key genera (Figure 3A). *Bifidobacterium*, initially dominant, decreased considerably in BCBF24 and BCL7524. However, its abundance remained higher in BCBF24, highlighting the prebiotic potential of this formulation, as *Bifidobacterium* is known to benefit gut health through the production of lactate and acetate [34,35]. These genus-level shifts align with other in vitro studies that demonstrate the targeted modulation of gut microbiota by specific substrates and probiotics [23,26].

*Segatella* and *Bacteroides* also showed clear declines in the synbiotic beverage falling to below 1% and 7% at 38 h, respectively. This implies a competitive exclusion of species such as *Bifidobacterium adolescentis*, *B. longum*, *Bacteroides uniformis*, and *Segatella copri*. The collective “Others” category also decreased in the synbiotic milk at later time points, indicating a general reduction in microbial diversity under these conditions. This reduction in diversity likely stems from the selective pressure exerted by metabolic by-products and nutrient availability in the synbiotic fermenters, which fostered a few dominant genera while suppressing others [36].

In contrast, the soymilk powder supported the growth of *Bacteroides*, which increased from 5% at 12 h to 20% at 38 h. This growth was largely due to the proliferation of *Bacteroides uniformis*, highlighting the specific capacity of the soymilk matrix to promote this particular species. The increase in *Bacteroides*, and specifically *B. uniformis*, in the soymilk group demonstrates the substrate-specific effects on gut microbiota composition, consistent with previous findings showing that different plant-based products can selectively enrich certain beneficial bacteria [35].

### 3.4. Genus-Level Gut Microbiota Abundance and SCFA Production

The relative abundance of SCFA production is shown in Table 1. Overall, the synbiotic beverage fermented with *L. plantarum* 75 and the soymilk sample produced significantly higher levels of all quantified SCFAs, except acetic acid, compared with BCBF24 treatment. This suggests that BCL7524 supported enhanced fermentative metabolism, producing SCFAs that could be further evaluated in vivo to determine their physiological relevance.

**Figure 4:**
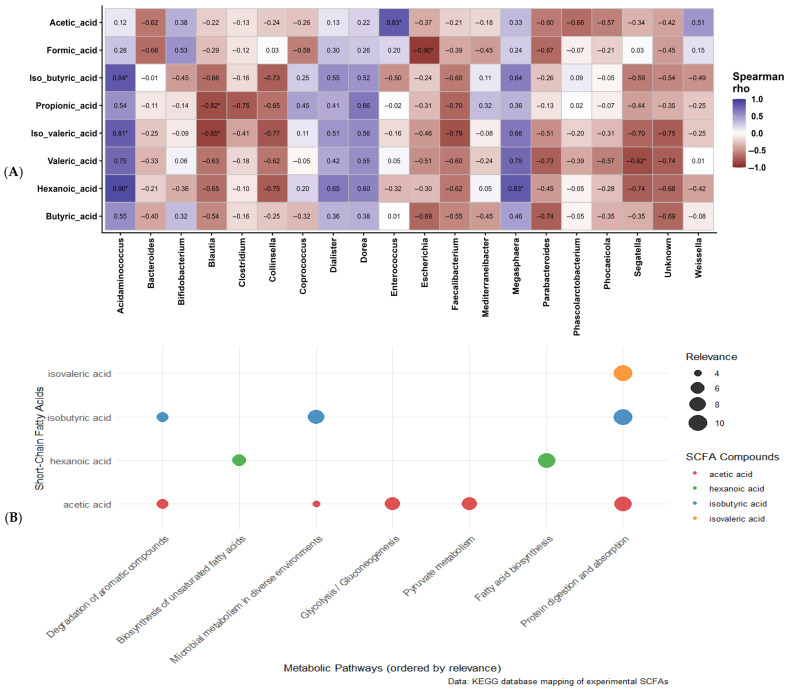
(**A**) Spearman correlation heatmap between genus-level gut microbiota and short-chain fatty acid (SCFA) production across all substrates and fermentation time points. The colour intensity and scale bar represent the correlation coefficient (r_s_), ranging from −1 (strong negative correlation, blue) to +1 (strong positive correlation, red); * indicates that the observed correlation is significant (*p* < 0.05), and (**B**) KEGG enrichment of metabolic pathways associated with increased SCFA production.

**Figure 5:**
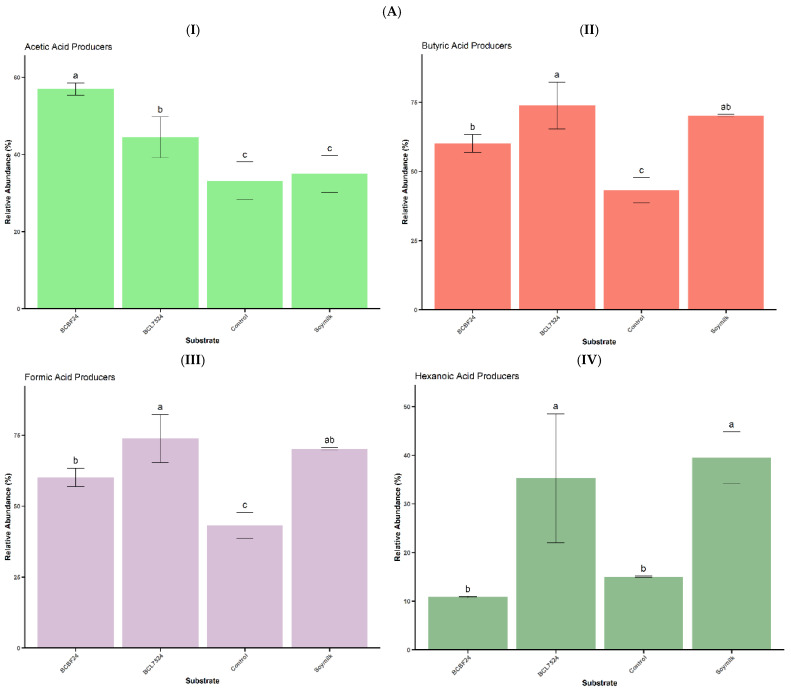

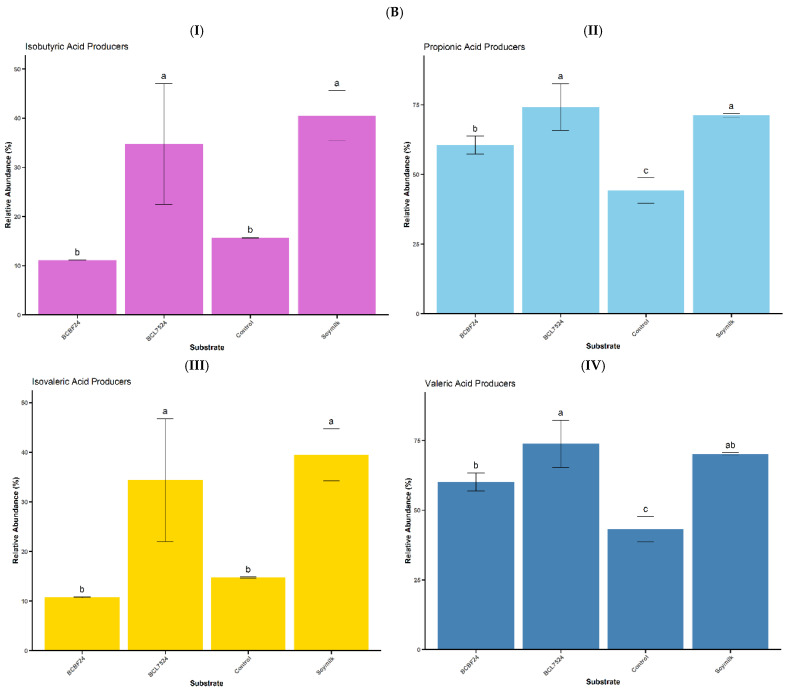
(**A**) Relative abundance (%) of short-chain fatty acid (SCFA)-producing bacterial genera following in vitro colonic fermentation of the different substrates. Bars assigned different superscript letters indicate a significant difference (*p* < 0.05). (**I**) Acetic acid producers (**II**) butyric acid producers (**III**) formic acid producers (**IV**) hexanoic acid producers. Treatments: Control (saline buffer only), BCBF24 (sprouted Bambara groundnut–cowpea milk fermented with *Bifidobacterium animalis* subsp. *lactis* BB-12), BCL7524 (sprouted Bambara groundnut–cowpea milk fermented with *Lactiplantibacillus plantarum* 75), inoculum and soymilk. (**B**) Relative abundance (%) of short-chain fatty acid (SCFA)-producing bacterial genera following in vitro colonic fermentation of the different substrates. Bars assigned different superscript letters indicate a significant difference (*p* < 0.05). (**I**) Isobutyric acid producers (**II**) propionic acid producers (**III**) isovaleric acid producers (**IV**) valeric acid producers. Treatments: Control (saline buffer only), BCBF24 (sprouted Bambara groundnut–cowpea milk fermented with *Bifidobacterium animalis* subsp. *lactis* BB-12), BCL7524 (sprouted Bambara groundnut–cowpea milk fermented with *Lactiplantibacillus plantarum* 75), inoculum and soymilk.

A Spearman correlation analysis was conducted to examine relationships between genus-level gut microbiota and SCFA production (Figure 4A). The results showed that *Megasphaera*, along with *Acidaminococcus* (a *Bacillota* genus), exhibited significant (*p* < 0.05) positive correlations with hexanoic acid. Although *Acidaminococcus* further showed significant (*p* < 0.05) positive correlations with isobutyric and isovaleric acids, *Megasphaera* showed positive correlations with these SCFAs without significant differences. These associations likely reflect cross-feeding interactions rather than direct proteolysis by *Megasphaera*, as this genus primarily utilises lactate and acetate [31,37]. KEGG pathway mapping (Figure 4B) supported this interpretation, as hexanoic acid was linked to fatty acid biosynthesis pathways consistent with de novo synthesis through microbial chain elongation. In this process, *Megasphaera* likely used lactate and acetate via reverse β-oxidation [37], a pattern consistent with its known role in lipid metabolism [37,38]. This metabolic versatility explains *Megasphaera*’s correlation with a broad range of SCFAs, including butyric, propionic, and valeric acids, generated through core fermentation pathways. These findings are corroborated by the significantly (*p* < 0.05) higher abundance of SCFA-producing taxa in BCL7524 (Figure 5A,B), which fostered a dominant *Megasphaera* population.

KEGG mapping indicated that isobutyric and isovaleric acids were linked to the protein digestion pathway, suggesting that proteolysis of the sprouted legume matrix by other community members generated the branched-chain intermediates required for branched-chain fatty acid (BCFA) production. Acetic acid also appeared in several of the mapped pathways; it functions as a central straight-chain metabolite that supports multiple microbial processes. Its co-occurrence with isobutyric acid across several pathways therefore suggests coordinated processing of protein- and carbohydrate-derived substrates within the community. Their presence in the aromatic compound degradation pathway may also reflect a breakdown of complex plant phenolics from the sprouted legume matrix (Figure S2).

In contrast, *Bifidobacterium* showed a non-significant positive correlation with acetic, butyric and formic acids. Other LAB genera, such as *Weissella*, showed a moderate positive correlation with acetic acid, while *Enterococcus* showed a significant (*p* < 0.05) positive correlation with acetic acid. These correlations with acetic acid correspond with the significant levels of acetic acid-producing taxa in the BCBF24 treatment (Figure 5A(I)). The recurrent mapping of acetic acid across multiple metabolic pathways (Figure 4B) highlights its central role as a metabolic intermediate, readily available for cross-feeding. This specialised niche, focused on acetate production rather than a broad SCFA spectrum, contrasts with the metabolically versatile *Megasphaera* consortium.

Several genera, including *Blautia*, *Escherichia*, and *Segatella*, showed significant (*p* < 0.05) negative correlations with propionic, isovaleric, formic, and valeric acids. These inverse relationships suggest competitive exclusion or distinct substrate preferences that do not support the production of these key metabolites [39]. This highlights how fermentable substrates in the synbiotic beverage shape both the SCFA profile and the microbial consortia responsible for its production.

## 4. Conclusions

This study demonstrates, for the first time to our knowledge, that synbiotic beverages from fermented, sprouted Bambara groundnut and cowpea create distinct selective niches that modulate gut microbial ecology and SCFA production. Using an in vitro colonic model, we showed that the synbiotic beverage fermented with *L. plantarum* enriched *Megasphaera* and a broad SCFA profile. Although KEGG analysis mapped hexanoic acid to fatty acid biosynthesis pathways, the combined SCFA pattern is consistent with microbial chain-elongation activity contributing to hexanoate formation. This highlights a key metabolic route in the valorisation of sprouted legumes. In contrast, the beverage fermented with *B. lactis* promoted a diverse community specialised in acetate production. While soymilk powder was included as a non-synbiotic control substrate, a limitation of this study is the absence of a non-fermented sprouted-seed beverage control. In addition, the study used faecal samples from only three donors, which limits the generalisability of the microbial responses observed. Future studies incorporating a non-fermented sprouted-seed control, together with a larger and more diverse donor cohort and subsequent in vivo validation, will be essential to clarify the relative contributions of substrate composition and fermentation to the microbial and metabolic responses observed and to strengthen the generalisability of the findings. In summary, this work highlights that germination and lactic acid bacteria fermentation can enhance substrate availability for saccharolytic and proteolytic bacteria within an in vitro model. This provides preliminary insights that could guide future research on legume-based synbiotic formulations and their potential use in functional food development.

## Figures and Tables

**Table 1:** Concentrations of short-chain fatty acids (SCFA, mM) before and after in vitro colonic fermentation of sprouted Bambara groundnut–cowpea milk fermented with *Bifidobacterium animalis* subsp. lactis or *Lactiplantibacillus plantarum* 75, and soymilk.

		Acetic Acid	Formic Acid	Isobutyric Acid	Propionic Acid	Isovaleric Acid	Valeric Acid	Hexanoic Acid	Butyric Acid
Control	Upper	0	0	0	0	0	0	0	0
	C12	5.49 ^a^ ± 0.03	0.32 ^a^ ± 0.02	0.18 ^a^ ± 0.02	3.78 ^a^ ± 0.22	0.08 ^a^ ± 0	1.33 ^a^ ± 0.47	0.11 ^a^ ± 0	0.91 ^a^ ± 0.38
	C24	6.11 ^ab^ ± 1.23	0.35 ^a^ ± 0.04	0.16 ^a^ ± 0.02	3.78 ^a^ ± 0.29	0.07 ^a^ ± 0.01	0.84 ^a^ ± 0.01	0.1 ^a^ ± 0.01	0.66 ^a^ ± 0.06
	C38	6.52 ^ab^ ± 1.13	0.43 ^a^ ± 0.02	0.22 ^a^ ± 0.09	4.14 ^ab^ ± 0.88	0.11 ^a^ ± 0.04	0.91 ^a^ ± 0.25	0.17 ^a^ ± 0.07	0.72 ^a^ ± 0.01
BCL7524	Upper	0	0	0	0	0	0	0	0
	C12	14.09 ^bc^ ± 2.72	4.84 ^b^ ± 0.45	0.77 ^b^ ± 0.07	6.96 ^abcd^ ± 0.56	1.16 ^b^ ± 0.1	10.38 ^c^ ± 0.57	2.67 ^b^ ± 0.82	12.36 ^d^ ± 0.88
	C24	18.36 ^cd^ ± 1.74	4.78 ^b^ ± 0.14	1.64 ^c^ ± 0.02	6.87 ^abc^ ± 0.33	2.07 ^c^ ± 0.24	11.30 ^c^ ± 0.51	8.45 ^c^ ± 0.29	10.51 ^d^ ± 3.73
	C38	18.99 ^cd^ ± 1.75	1.14 ^a^ ± 0.04	2.73 ^e^ ± 0.1	9.74 ^cde^ ± 0.54	3.48 ^d^ ± 0.05	14.55 ^d^ ± 0.91	13.92 ^d^ ± 0.14	9.74 ^cd^ ± 3.24
BCBF24	Upper	0	0	0	0	0	0	0	0
	C12	17.8 ^cd^ ± 2.67	1.54 ^a^ ± 0.17	0.14 ^a^ ± 0.02	6.92 ^abc^ ± 0.44	0.14 ^a^ ± 0.03	1.82 ^a^ ± 0.09	0.09 ^a^ ± 0.01	2.25 ^abc^ ± 0.65
	C24	20.24 ^cd^ ± 2.84	1.6 ^a^ ± 0.18	0.1 ^a^ ± 0.01	8.03 ^cde^ ± 0.26	0.09 ^a^ ± 0.01	1.57 ^a^ ± 0.05	0.01 ^a^ ± 0.01	2.48 ^abc^ ± 0.08
	C38	22.69 ^d^ ± 1.01	1.41 ^a^ ± 0.11	0.14 ^a^ ± 0	8.89 ^cde^ ± 0.38	0.12 ^a^ ± 0.01	1.97 ^a^ ± 0.08	0.1 ^a^ ± 0.01	1.88 ^ab^ ± 0.48
Soymilk	Upper	0	0	0	0	0	0	0	0
	C12	14.1 ^bc^ ± 1.74	7.79 ^c^ ± 1.25	1.04 ^b^ ± 0.06	7.38 ^bcd^ ± 0.81	1.4 ^bc^ ± 0.05	6.82 ^b^ ± 0.56	3.42 ^b^ ± 0.22	13.03 ^d^ ± 3.83
	C24	16.2 ^cd^ ± 4.11	1.68 ^a^ ± 0.08	2.2 ^d^ ± 0.38	11.24 ^e^ ± 2.47	3.06 ^d^ ± 0.57	9.81 ^bc^ ± 2.08	7.60 ^c^ ± 1.08	7.05 ^abcd^ ± 1.73
	C38	12.92 ^abc^ ± 0.47	0.52 ^a^ ± 0.05	2.62 ^de^ ± 0.04	10.44 ^de^ ± 0.7	3.28 ^d^ ± 0.18	10.04 ^c^ ± 1.21	8.08 ^c^ ± 0.54	8.82 ^bcd^ ± 0.97

Means assigned different superscript letters per column indicate a significant difference (*p* < 0.05). C12 (colonic fermentation for 12 h), C24 (colonic fermentation for 24 h), and C38 (colonic fermentation for 38 h). Treatments: Control (saline buffer only), BCBF24 (sprouted Bambara groundnut–cowpea milk fermented with *Bifidobacterium animalis* subsp. *lactis* BB-12), BCL7524 (sprouted Bambara groundnut–cowpea milk fermented with *Lactiplantibacillus plantarum* 75), and soymilk.

## Data Availability

The original contributions presented in this study are included in the article/Supplementary Materials. Further inquiries can be directed to the corresponding author.

## Supplementary Materials

The following supporting information can be downloaded at: https://www.mdpi.com/article/10.3390/foods15071141/s1, Figure S1: Experimental design; Figure S2: Total phenolic content (TPC) and ferric reducing antioxidant power (FRAP) of the beverage powders before and after in vitro digestion; Table S1: Total dietary fibre content of the initial beverage and survival of lactic acid bacteria (LAB) before and after in vitro digestion; Table S2: Short-chain fatty acid identification and quantification parameters.
