# Supplementary material for: Probiotic Lactic Acid Bacteria-Fermented Beverages from Bambara Groundnut and Cowpea Sprouts Modulate Gut Microbiota and Short-Chain Fatty Acids
Source: Foods. 2026 Mar 26;15(7):1141. doi: 10.3390/foods15071141 (PMC13072960; doi:10.3390/foods15071141)
Supplement: Supplementary file 1 [file foods-15-01141-s001.zip › foods-4152750-supplementary.pdf]

foods

Supplementary Materials

Probiotic Lactic Acid Bacteria Fermented Beverages from Bambara Groundnut and Cowpea Sprouts Modulate Gut Microbiota and Short-Chain Fatty Acids

Nobahle Pretty Cele[1], Yusuf Olamide Kewuyemi[1], Oladipupo Adiamo[2], Eshetu Mulisa Bobasa[2], Jiale Zhang[2], Maral Seididamyeh[2], Yasmina F. Sultanbawa[2] and Dharini Sivakumar [1,2]*

1 Phytochemical Food Network, Department of Crop Sciences, Tshwane University of Technology, Pretoria, South Africa; zamandoccy24@gmail.com (N.P.C.); kewuyemiyo@tut.ac.za (Y.O.K.)

2 Centre for Nutrition & Food Sciences, Queensland Alliance for Agriculture and Food Innovation, The University of Queensland, Brisbane, QLD 4108, Australia; o.adiamo@uq.edu.au (O.A.); e.bobasa@uq.edu.au (E.M.B.); jiale.zhang1@student.uq.edu.au (J.Z.); s.maral@uq.edu.au (M.S.); y.sultanbawa@uq.edu.au (Y.F.S.)

* Correspondence: sivakumard@tut.ac.za

foods MDPI

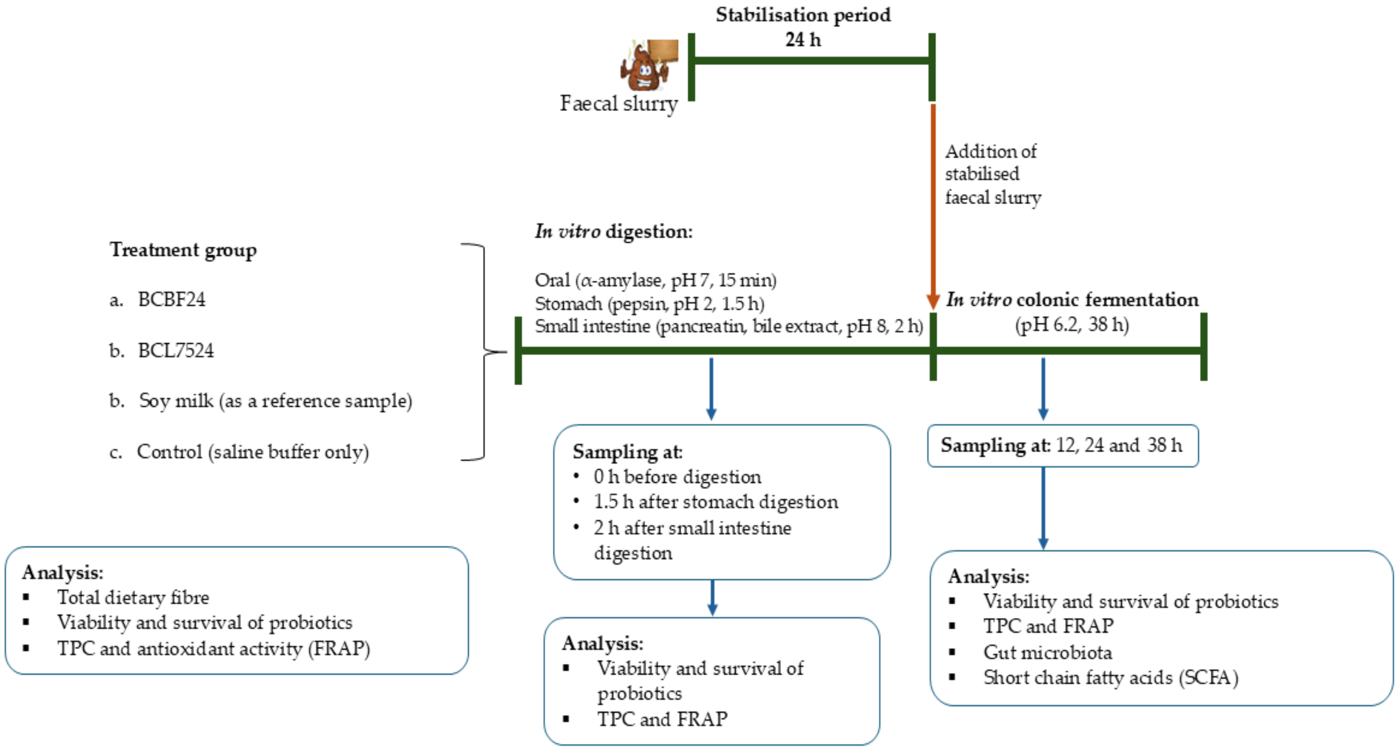

Figure S1: Experimental design

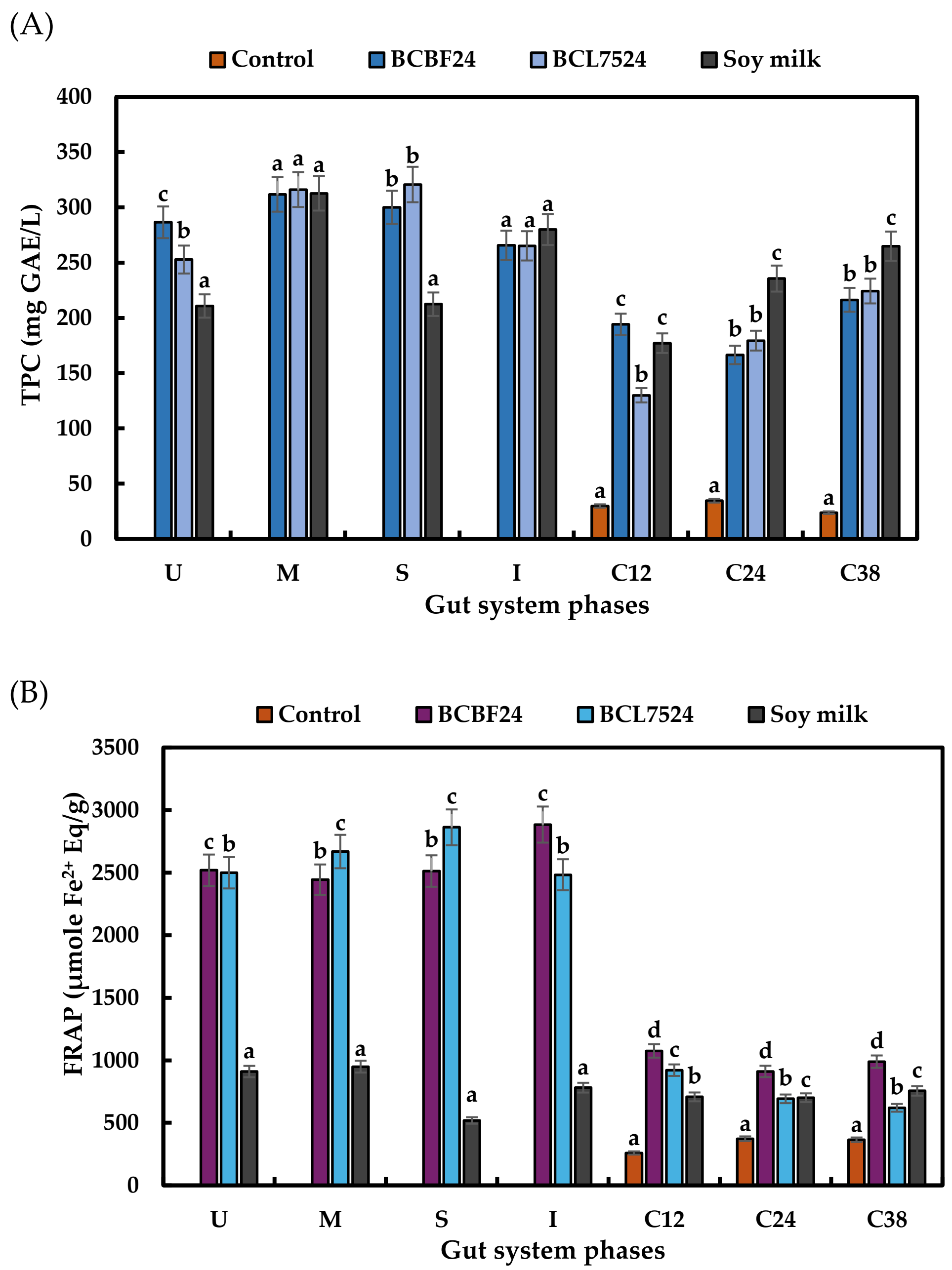

Figure S2: Total phenolic content (TPC) (A) and ferric reducing antioxidant power (FRAP) (B) of the beverage powders before and after *in vitro* digestion. Bars assigned different superscript letters indicate a significant difference ($p < 0.05$). C12 (Colonic fermentation for 12 h), C24 (Colonic fermentation for 24 h), C38 (Colonic fermentation for 38 h), U (undigested), M (mouth phase), S (stomach phase), and I (small intestine), mg GAE/g (milligrams of gallic acid equivalent per gram) and µmole Fe^{2+}Eq/g (micromoles of Fe^{2+} equivalent per gram). Treatments: Control (saline buffer only), BCBF24 (sprouted Bambara groundnut-cowpea milk fermented with *Bifidobacterium animalis* subsp. *lactis* BB-12), BCL7524 (sprouted Bambara groundnut-cowpea milk fermented with *Lactiplantibacillus plantarum* 75), and soymilk.

Table S1: Total dietary fibre content of the initial beverage and survival of lactic acid bacteria (LAB) before and after *in vitro* digestion

	**Control**	**BCBF24**	**BCL7524**	**Soymilk**	**FS**
**Total dietary fibre (g/100 g)**	nd	$17.58^{a} \pm 0.17$	$17.50^{a} \pm 0.56$	$19.91^{b} \pm 0.99$	nd
**LAB survival (Log CFU/mL)**					
Gut system phases					
Undigested	nd	8.15^{a}±0.57	8.29^{a}±0.57	nd	nd
Mouth	nd	8.03^{a}±0.57	8.22^{a}±0.57	nd	nd
Stomach	nd	6.79^{a}±0.57	6.92^{a}±0.57	nd	nd
Small intestine	nd	6.44^{a}±0.57	6.57^{a}±0.57	nd	nd
Colon	nd	nd	nd	nd	6.22
C12	7.31^{a}	8.45^{b}±0.57	8.46^{b}±0.57	7.31^{a}±0.57	nd
C24	6.27^{a}	7.31^{c}±0.57	7.33^{c}±0.57	6.62^{ab}±0.57	nd

Means assigned different superscript letters indicate a significant difference ($p < 0.05$). C12 (Colonic fermentation for 12 h), C24 (Colonic fermentation for 24 h), and nd (not determined). Treatments: Control (saline buffer only), BCBF24 (sprouted Bambara groundnut-cowpea milk fermented with *Bifidobacterium animalis* subsp. *lactis* BB-12), BCL7524 (sprouted Bambara groundnut-cowpea milk fermented with *Lactiplantibacillus plantarum* 75), FS (Faecal sample), and soymilk.

Table S2: Short chain fatty acid identification and quantification parameters

	Compound	Rt	Quantification ion	Reference ions
1	Formic acid	9.0	136.11	108.07, 91.06
2	Acetic acid	9.6	150.02	91.07, 50.07
3	Isobutyric acid	10.2	178.05	91.02, 71.09
4	Propionic acid	10.3	164.08	108.05, 57.07
5	Butyric acid	11.4	178.12	160.07, 71.04
6	Isovaleric acid	11.5	192.08	85.07, 57.12
7	Valeric acid	12.2	192.10	85.09, 57.13
8	Hexanoic acid	13.3	206.12	188.14, 92.11

Rt (Retention time)
